# Quality of life after hip fracture: a 12-month prospective study

**DOI:** 10.7717/peerj.9215

**Published:** 2020-06-16

**Authors:** Francisco Javier Amarilla-Donoso, Raul Roncero-Martin, Jesus Maria Lavado-Garcia, Rosaura Toribio-Felipe, Jose Maria Moran-Garcia, Fidel Lopez-Espuela

**Affiliations:** 1 Department of Nursing, Hospital Campo Arañuelo de Navalmoral de la Mata, Navalmoral de la Mata, Spain; 2 Department of Nursing, Nursing and Occupational Therapy College, University of Extremadura, Cáceres, Spain; 3 Department of Nursing, Hospital Virgen del Puerto, Plasencia, Spain

**Keywords:** Geriatric care, Hip fracture, Health-related quality of life

## Abstract

**Background:**

Hip fracture is an important and frequent health problem worldwide. To date, there are still limited studies focused on the analysis of health-related quality of life (HRQOL) after a hip fracture in the Spanish population, especially with long-term follow-up.

**Objective:**

To determine the HRQOL at 12 months after hip fracture and to identify potential factors associated with HRQOL.

**Design:**

Prospective observational study.

**Setting:**

Traumatology units of two university hospitals in province Cáceres (Spain).

**Participants:**

A total of 224 patients were admitted to the unit and required immediate surgery due to a hip fracture.

**Methods:**

HRQOL was measured with the EuroQol-5D questionnaire (EQ-5D) and the SF-12 Health Survey.

**Results:**

Scores from the visual analog scale EQ-5D decreased significantly (*p* < 0.001) from 72.8 at baseline to 48.3 after 1 month, to 48.2 after 6 months and to 46.1 after 12 months. The EQ-5D index score showed a similar significant reduction (*p* < 0.001) from 0.6 to 0.1, 0.3 and 0.3, respectively. Values of the physical component summary (PCS-12) significantly decreased (*p* < 0.001) from 38.6 at baseline to 31.0, 33.1 and 33.5. The mental component summary (MCS-12) decreased from 46.5 to 44.8 after 6 months (*p* = 0.022) and 44.3 after 12 months (*p* = 0.005). Factors potentially associated with HRQOL at 12 months after hip fracture were depression status after 12 months (*B* = 0–1.876; 95% CI [−2.409 to −1.343]; *p* < 0.001), functional ambulation classification after 12 months (B = −12.133; 95% CI [−17.970 to −6.297]; *p* < 0.001), EQ-5D VAS at baseline (*B* = 0.223; 95% CI [0.115–0.330]; *p* < 0.001), and age (*B* = −0.323; 95% CI [−0.594 to −0.053; *p* = 0.015).

**Conclusions:**

Patients experience a significant impairment in HRQOL H after a hip fracture, especially in self-care, pain/discomfort, usual activities, mobility and anxiety/depression. The decline in the HRQOL is effective the first month and lasts at least 12 months after the surgical intervention.

## Introduction

Hip fracture is an important health problem due to its incidence, as well as its important physical (Marks, 2010), social and economic implications (Carpintero et al., 2014), representing an important challenge for health care in the future, since the growing population of hip fractures has increasingly complex medical, social and rehabilitation needs (Baker et al., 2014). There is an important association of hip fractures with age, as most hip fractures occur in people older than 65 years. Since life expectancy continues to increase, it is likely that the incidence of fragility fractures in Spain will also increase by almost 30% in 2030 (International Osteoporosis Foundation, 2019).

Hip fractures affect one in three women and one in five men aged 50 and over. In 2017, there were 73,381 discharges due to hip fracture, of which 71% were women (INE, 2019). Hip fracture is associated with an excess of mortality, morbidity and disability (Abrahamsen et al., 2009). Mortality after a hip fracture ranges between 8.4% and 36% (Abrahamsen et al., 2009; Roche et al., 2005; Ekegren et al., 2016; Caeiro et al., 2017) and is 5–8 times higher in the 3 months following the fracture. This increased risk persists even after 10 years (Haentjens et al., 2010).

Approximately one in three women will suffer a hip fracture during their lifetime, especially from falls or as a consequence of osteoporosis (Chami et al., 2006). From an economic perspective of health, the burden of disease covers both the cost related to the disease and its consequences on morbidity (i.e., quality of life and survival) (Borgström et al., 2013). It is estimated that the average direct costs of caring for a hip fracture in Spain are at 8,400 euros, with global figures that reach between 300 and 860 million euros (Health Information Institute, 2010). In addition, hip fractures usually require a surgical intervention followed by postoperative rehabilitation to recover the prefracture function level. Hip fractures are the cause of 8% of visits to emergency services and 42% of hospital admissions related to falls (Ruths et al., 2017). Between 50% and 71% of people who experience a hip fracture will recover the levels prior to the fracture by 1 year later (Vergara et al., 2014; Dyer et al., 2016), with the consequent decline in their independence, so that up to one in four patients will be institutionalized.

Rehabilitation programs with physical exercises have been shown to have a positive effect on functional abilities (Lima et al., 2016); however, it is common that, despite following an adequate rehabilitation treatment, recovery is incomplete, and reduced mobility, imbalance, lack of confidence or fear of falling after hip fracture remain (Shumway-Cook et al., 2005; Portegijs et al., 2012). In fact, 25–75% of people who can walk independently before the fracture will become dependent after 12 months (Tang et al., 2017).

Hip fracture has been associated with a profound deterioration in the health-related quality of life (HRQOL) of the patient (González-Zabaleta et al., 2015; Peeters et al., 2016). There are innumerable studies and reports aimed at knowing the impact of the fracture through objective clinical and socioeconomic indicators, obviating the self-perception of the patient about their own functional development. However, this traditional view is changing, and an increasing number of studies and reports incorporate subjective indicators based on the self-perception of the patient as a complement to traditional indicators to evaluate clinical interventions. To our knowledge, there are still limited studies focused on the analysis of HRQOL after a hip fracture in the Spanish population, especially with long-term follow-up (Støen et al., 2014; Gjertsen et al., 2016; Alexiou et al., 2018). Given the importance of HRQOL in successful aging (Li et al., 2014), it is necessary to adequately understand the impact of a hip fracture on HRQOL. Therefore, the objective of this study was to determine HRQOL after 12 months of hip fracture and to identify potential factors associated with HRQOL.

The self-assessment of the quality of life is highly influenced by personal experience and cultural constraints.

## Methods

### Design

A prospective observational study was conducted in the traumatology units of two university hospitals in the province of Cáceres (Spain) between June 2015 and June 2017.

Consecutive sampling was performed. The inclusion criteria were patients over the age of 65-year-old admitted with a primary diagnosis of hip fracture who underwent emergency surgery for surgical reduction of the fracture; patients without cognitive impairment and who were not terminally ill; and those without linguistic barriers that would prohibit them from understanding the questionnaires.

All patients included in the study were treated according to standard clinical practices. Written informed consent was required to participate in the study. The procedures were compliant with the Declaration of Helsinki and were approved by the Clinical Research Ethics Committee of Cáceres (Spain) (Ref. 15/0145). This acceptance by the ethics committee allowed the development of the study in the two hospitals where it was carried out.

### Studied variables

A questionnaire containing the following variables was drawn up for data collection: clinical, sociodemographic and economic data, personal history, standard treatment, clinical variables of functional dependance (Barthel Index, Lawton-Brody Scale), and social-familial assessment (Gijón Assessment Scale), HRQOL variables (EuroQol-5D), days of hospitalization, delay in surgical intervention, destination after discharge, functional ambulation capacity, presence of complications and polymedication. The Charlson Comorbidity Index (CCI) was used as a method to quantify the number of chronic disorders and their severity. The American Society of Anesthesiologists (ASA) scale was used to classify physical fitness. The questionnaire was completed at the time of admission and during the follow-up visit (1 month, 6 months and 12 months). Data were obtained through personal interviews with patients and a review of the hospital medical records. To ascertain the baseline situation, the participants were interviewed about their functional situation and quality of life two weeks before the fracture, and this information was identified as the baseline condition.

Health-related quality of life was assessed using the EuroQol-5D questionnaire (Devlin & Brooks, 2017) widely used as a generic measure of health for clinical and economic appraisal tool. The EuroQol-5D is a descriptive system with five domains (mobility, self-care, regular activities, pain/discomfort and anxiety/depression) divided into three levels of severity, from which a weighted score is derived based on cultural and national differences. This system also includes a visual analog scale (EQ-5D VAS) (EuroQol Research, Foundation, 2017) defined by a 20 cm vertical scale at either end of which are the extreme expressions of self-perceived state of health ranging from 0 (worst health) to 100 (best health). Responses to the state of health classification system were converted to an overall score following the instructions for the application of the questionnaire, tariff calculation explanations and recommendations on the presentation of results for the Spanish population (Badia et al., 1999). The HRQOL was also measured with the SF-12 Health Survey, consisting of 8 domains divided into a physical component summary score (PCS, including general health, physical functioning, role physical and body pain) and a mental component summary score (MCS, including vitality, social functioning, role emotional and mental health) (Gandhi et al., 2001; Vilagut et al., 2008). Each component can be scored from 0 (lowest level of health) to 100 (highest level of health).

The ability to perform basic activities of daily living (BADL) was assessed using the Barthel Index (Mahoney & Barthel, 1965). This scale evaluates ten elements (feeding, bathing, grooming, dressing, bowels, bladder, toilet use, transfers, mobility and stairs). A total score between 0 and 20 suggests total dependance for the performance of BADL: 21 to 60, severe dependance; 61 to 90, moderate dependance; 91 to 99, mild dependance; and 100, independence (Shah, Vanclay & Cooper, 1989). The ability to perform instrumental activities of daily living (IADL) was assessed using the Lawton and Brody scale (Lawton & Brody, 1969), which assesses eight items (ability to use the telephone, shopping, food preparation, housekeeping, laundry, mode of transportation, responsibility for own medications and ability to handle finances). Taking gender differences into account, total dependency was categorized as 0 in men and 0–1 in women; severe as one in men, 2–3 in women; moderate as 2–3 in men and 4–5 in women; minor as four in men and 6–7 in women; and independent as five in men and eight in women. Ambulation capacity was determined through the use of functional ambulation categories (Holden, Gill & Magliozzi, 1986), a scale with six possible scores 0–5, where the lower the score is, the greater the dependance. A total score of 0–3 indicated that the patient was dependent or non-ambulatory; 4–5 suggested independence. Symptoms of depression in geriatric patients were detected using the Spanish version of the 15-point Geriatric Depression Scale (Yesavage et al., 1983; Martí et al., 2000). A score of 0–5 indicated no depression; 6–9 suggested possible depression, and ≥10 revealed an established depression. The socio-familiar situation was determined by the Gijon Socio-familiar Scale (Díaz, Domínguez & Toyos, 1993), which assesses five dimensions (family situation, economic situation, housing, social relations and social support network). A total score between 5 and 9 indicates a good or adequate social situation,10–14 indicates social risk, and ≥15 indicates a social problem.

Comorbidity was calculated using the CCI (Charlson et al., 1987). This index is a predictive model that assigns numerical values to different chronic pathologies, obtaining a final score for each individual patient by adding the partial values. We identified patients who took five or more medications daily for a period of more than 6 months as polymedicated.

### Statistical analysis

The descriptive analysis was performed by calculating the percentage of categorical variables and the mean together with the standard deviation (SD) of distributions in continuous variables: age, EQ-5D VAS, CCI, Barthel Index, IADL of Lawton and Brody, Yesavage Geriatric Depression Scale and Gijon Scale.

The analysis of paired groups (temporal points) was carried out with the Wilcoxon test for continuous variables (EQ-5D VAS, Barthel Index, IADL of Lawton and Brody, Yesavage Geriatric Depression Scale and Gijon Scale) and with the McNemar test for categorical variables. Changes in dimensions from the HRQOL were evaluated using the Cochran test.

The identification of independent factors potentially associated with the HRQOL (after 12 months) was performed by developing a stepwise multiple linear regression model, considering HRQOL as the dependent variable (measured with the EQ-5D index and EQ-5D VAS). The variables with significance indicated as *p* < 0.05 in the univariate analysis were included in the multivariate model. Independent variables showing collinearity were excluded from the model. The results from the model were regression coefficients (*B*) and respective 95% confidence intervals (95% CI). Statistical significance was established when *p* ≤ 0.05. Analyses were carried out with SPSS 20.0 software.

## Results

A total of 270 patients were admitted for hip fracture during the study period. Of these patients, three (1.1%) refused to participate in the study, 43 (15.92%) met one of the exclusion criteria, and two (0.8%) died before they could be recruited for inclusion in the study. A total of 224 patients were included in the study. The mortality during the first postdischarge month was five patients (2.2%).

The sociodemographic and clinical characteristics of the patients are shown in Table 1. The mean age of the study participants was 84.6 years (SD ± 6.1 years); the majority of patients were women (76.3%) and polymedicated (69.6%). A total of 64.3% of the patients suffered a trochanteric fracture, compared to 35.7% who suffered a neck fracture. Fracture reduction by intramedullary rods was the most common type of surgical procedure (66.2%). Most surgeries were performed under spinal anesthesia (79.5%). The time between hospital admission and surgery was 3.0 days (SD ± 2.8 days), and the hospital stay was 5.3 days (SD ± 1.2 days). Charlson’s Comorbidity Index at the time of surgery was 5.3 (SD ± 1.2).

**Table 1:** Demographic and clinical characteristics of patients.

	Total patients (*n* = 224)
Gender *n* (%)	
Female	171 (76.3)
Male	53 (23.7)
Age mean years (SD)	84.6 (6.1)
Groups *n* (%)	
<85 years	106 (47.3)
≥85 years	118 (52.7)
Study level *n* (%)	
No studies	85 (37.9)
Primary	130 (58.0)
Secondary	6 (2.7)
University	3 (1.3)
Living status *n* (%)	
Living alone	57 (25.4)
Living in couple	61 (27.2)
Living with relatives	62 (27.7)
Supervised flat	7 (3.1)
Residency	37 (16.5)
Clinical history *n* (%)	
Hypertension	166 (74.1)
Diabetes mellitus	65 (29.0)
Dyslipidemia	76 (33.9)
Osteoporosis	21 (9.4)
Previous hip fracture *n* (%)	18 (8.0)
Polymedicated *n* (%)	156 (69.6)
Charlson comorbidity index mean (SD)	5.3 (1.2)
Type of fracture *n* (%)	
Neck	80 (35.7)
Trochanter	144 (64.3)
Type of surgical intervention *n* (%)	
Intramedullary nail	147 (66.2)
Hip replacement	75 (33.8)
Perioperative complications *n* (%)	
No	191 (85.3)
Yes	33 (14.7)
ASA PS for peri-operative risk *n* (%)	
I	1 (0.4)
II	70 (31.3)
III	136 (60.7)
IV	17 (7.6)
Destination after surgery *n* (%)	
Home	98 (43.8)
Institution	84 (37.5)
With relatives	40 (17.9)
Has fallen again during the follow-up *n* (%)	
Immediately after surgery	13 (5.9)
After 6 months	18 (8.6)
After 12 months	4 (2.0)
Has received rehabilitation *n* (%)	
Immediately after surgery	100 (45.7)
After 6 months	13 (6.2)
After 12 months	2 (1.0)
Exitus *n* (%)	
Baseline	2 (0.9)
After 1 month	5 (2.2)
After 6 months	16 (7.1)
After 12 months	19 (8.5)
Previous hip fracture *n* (%)	18 (8.0)
Time elapsed between hospital admission and intervention mean (SD)	3.02 (±2.8)
Time elapsed between hospital admission and discharge mean (SD)	5.3 (±1.2)

**Note:**

ASA PS, American Society of Anesthesiologists physical status.

**Figure 1:**
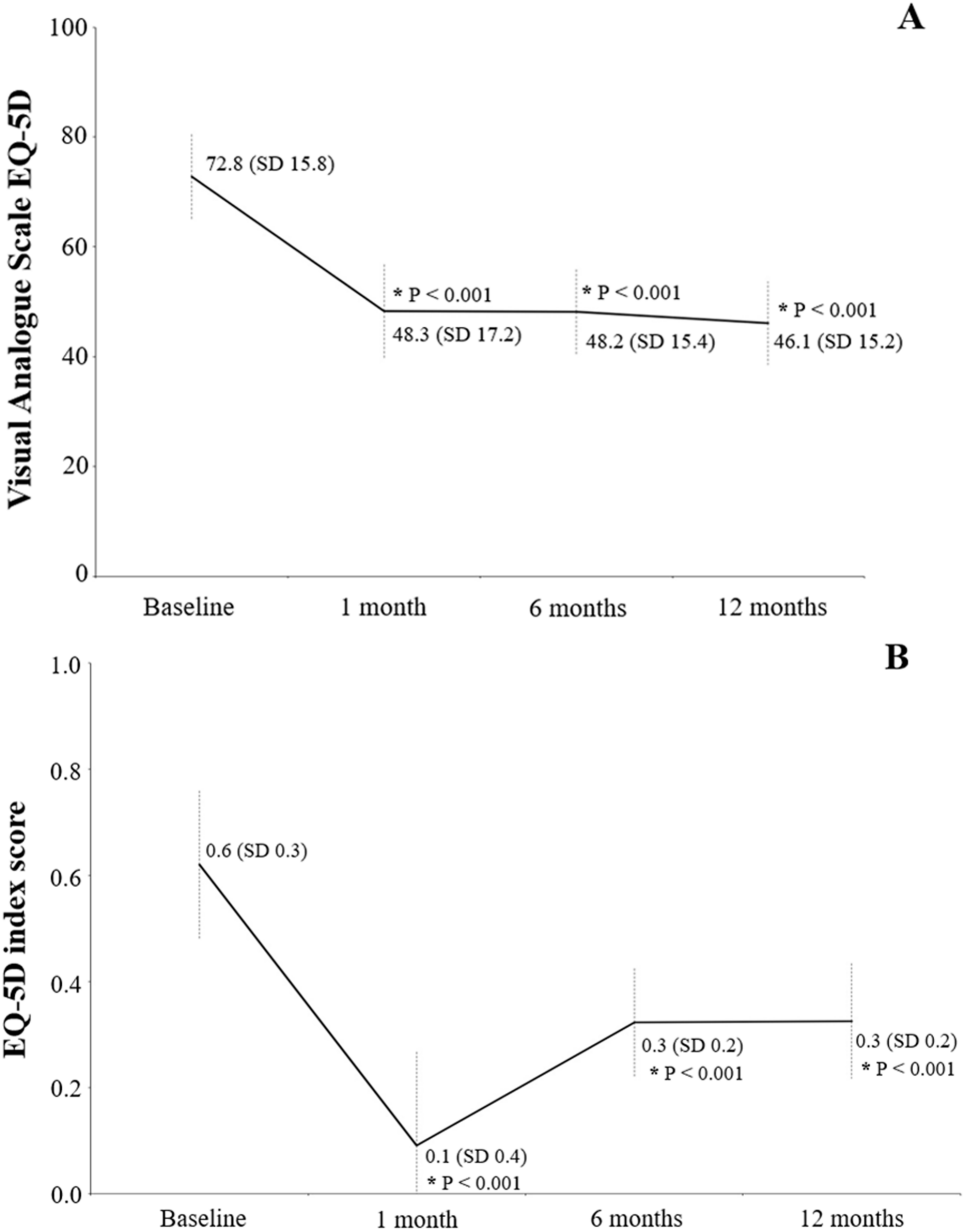
Health-related quality of life using the EQ-5D questionnaire: with the visual analog scale (A) and the EQ-5D index score (B). Values written in the graphic are the mean and the standard deviation (in parenthesis). An asterisk indicates a value significantly different from baseline.

**Figure 2:**
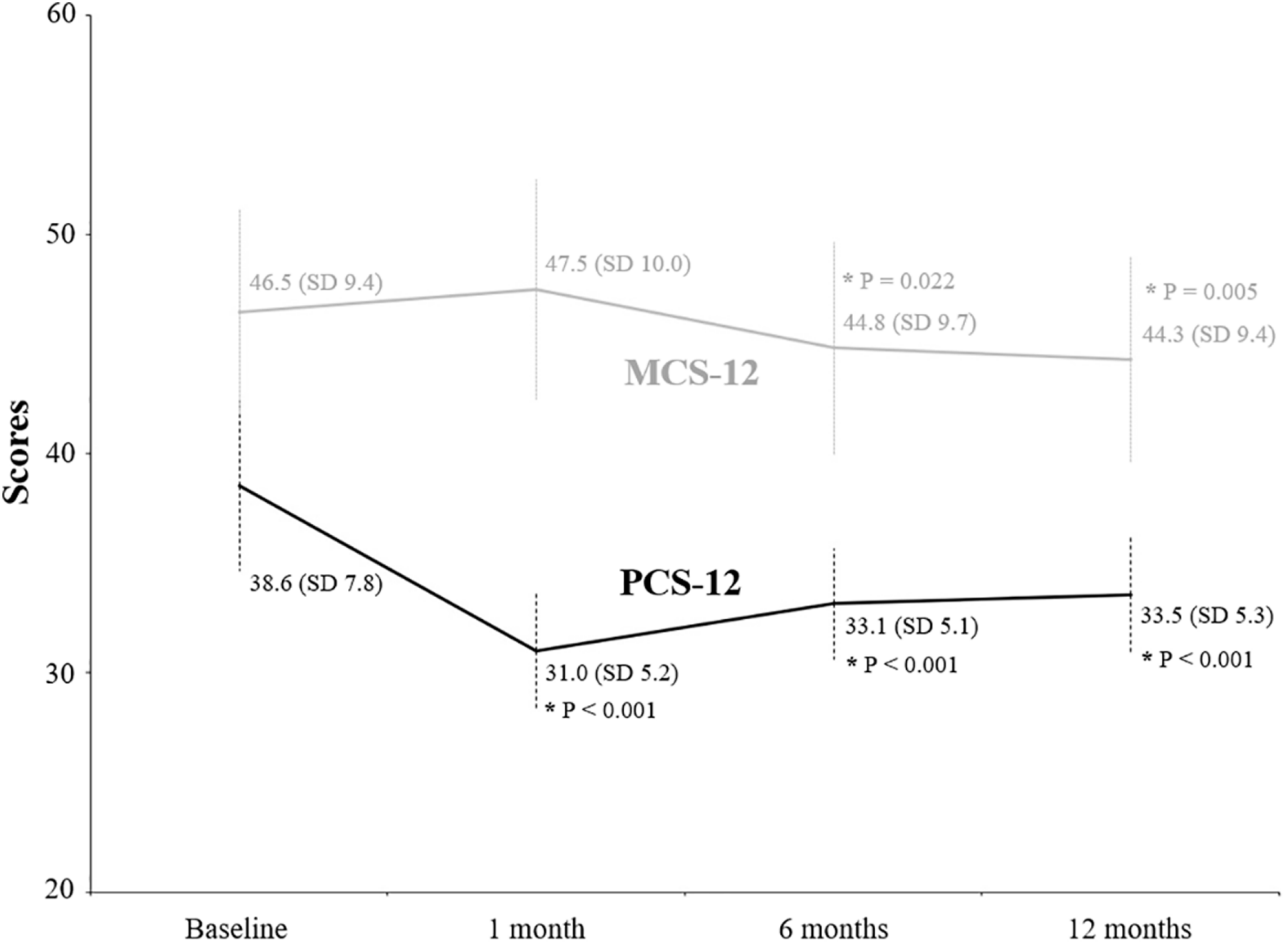
Evolution of scores from the physical and mental component summary through the follow-up period. Values written in the graphic are the mean and the standard deviation (in parenthesis). An asterisk indicates a value significantly different from baseline.

The evolution of clinical factors and the functional ability of patients during the follow-up period are shown in Table 2. The mean scores of IADL Lawton and Brody decreased significantly (*p* < 0.001) from 5.1 (SD 2.7) at baseline to 2.2 (SD 1.5), 2.3 (SD 1.9) and 2.3 (SD 1.9) after 1, 6 and 12 months, respectively. Similarly, the Barthel Index decreased significantly (*p* < 0.001) from 87.6 (SD 16.9) at baseline to 53.5 (SD 17.7), 58.9 (SD 19.5) and 59.1 (SD 19.6). The percentage of patients indicating not being able to walk independently (FAC) increased significantly (*p* < 0.001) from 42.8% at baseline to 99.1% after 1 month, to 95.2% after 6 months and to 91.2% after 12 months. The mean value of the Yesavage Geriatric Depression Scale also increased significantly (*p* < 0.001) from 3.2 (SD 3.7) at baseline to 5.6 (SD 3.3), 6.2 (SD 3.2) and 6.6 (SD 3.2), respectively. Scores from the EQ-5D VAS decreased significantly (*p* < 0.001) from 72.8 (SD 15.8) at baseline to 48.3 (SD 17.2) after 1 month, to 48.2 (SD 15.4) after 6 months and to 46.1 (SD 15.2) after 12 months (Fig. 1A). The EQ-5D index score showed a similar significant reduction (*p* < 0.001) from 0.6 at baseline to 0.1, 0.3 and 0.3, respectively (Fig. 1B). The values for PCS-12 significantly decreased (*p* < 0.001) from 38.6; SD 7.8 at baseline to 31.0 (SD 5.2) after 1 month, to 33.1 (SD 5.1) after 6 months and to 33.5 (SD 5.3) after 12 months (Fig. 2). Similarly, MCS-12 decreased from 46.5 (SD 9.4) at baseline to 44.8 (SD 9.7; *p* = 0.022) after 6 months and to 44.3 (SD 9.4; *p* = 0.005) after 12 months. Changes in the dimensions of the EQ-5D questionnaire are shown in Table 3. All dimensions showed significant changes from baseline after 1, 6 and 12 months, especially by increasing the perception of “some problems”.

**Table 2:** Evolution of clinical factors and the functional ability of patients during the follow-up period.

	Baseline		1 month			6 months			12 months		
	***n***	Value	***n***	Value	*p* With baseline	***n***	Value	*p* With baseline	***n***	Value	*p* With baseline
Lawton and Brody scale mean (SD)*	224	5.1 (2.7)	219	2.2 (1.5)	<0.001	208	2.3 (1.9)	<0.001	205	2.3 (1.9)	<0.001
Barthel index. mean (SD)*	224	87.6 (16.9)	219	53.5 (17.7)	<0.001	208	58.9 (19.5)	<0.001	205	59.1 (19.6)	<0.001
Functional Ambulation Classification: Do not walk independently *n* (%)^&^	222	95 (42.8)	219	217 (99.1)	<0.001	208	198 (95.2)	<0.001	205	187 (91.2)	<0.001
Yesavage’s geriatric depression scale mean (SD)*	224	3.2 (3.7)	219	5.6 (3.3)	<0.001	208	6.2 (3.2)	<0.001	205	6.6 (3.2)	<0.001
Gijon scale mean (SD)*	182	8.3 (2.0)	129	8.5 (1.9)	0.090	129	8.6 (1.8)	0.010	129	8.6 (1.9)	0.030

**Notes:**

* *p*-Value from Wilcoxon test.

& *p*-Value from McNemar test.

IADL, instrumental activities of daily living scale; SD, standard deviation.

**Table 3:** Changes in dimensions from the EQ-5D questionnaire through the follow-up period.

	Baseline	1 month		6 months		12 months	
	*n* (%)	*n* (%)	*p* With baseline*	*n* (%)	*p* With baseline*	*n* (%)	*p* With baseline*
Mobility			<0.001		<0.001		<0.001
No problems	96 (42.9)	1 (0.5)		8 (3.8)		9 (4.5)	
Some problems	127 (56.7)	215 (98.2)		197 (94.7)		189 (94.0)	
Severe problems	1 (0.4)	3 (1.4)		3 (1.4)		3 (1.5)	
Self-care			<0.001		<0.001		<0.001
No problems	134 (59.8)	4 (1.8)		23 (11.1)		24 (11.9)	
Some problems	74 (33.0)	118 (53.9)		118 (56.7)		112 (60.0)	
Severe problems	16 (7.1)	97 (44.3)		67 (32.2)		66 (32.7)	
Usual activities			<0.001		<0.001		<0.001
No problems	107 (47.8)	2 (0.9)		10 (4.8)		12 (5.9)	
Some problems	67 (29.9)	39 (17.8)		58 (27.9)		57 (28.2)	
Severe problems	50 (22.3)	178 (81.3)		140 (67.3)		133 (65.8)	
Pain/discomfort			<0.001		<0.001		<0.001
No problems	98 (43.8)	32 (14.6)		37 (17.8)		39 (19.3)	
Some problems	104 (46.4)	177 (80.8)		164 (78.8)		154 (76.2)	
Severe problems	22 (9.8)	10 (4.6)		7 (3.4)		9 (4.5)	
Anxiety/depression			<0.001		<0.001		<0.001
No problems	145 (64.7)	103 (47.0)		76 (36.5)		74 (36.6)	
Some problems	71 (31.7)	103 (47.0)		125 (60.1)		120 (59.4)	
Severe problems	8 (3.6)	13 (5.9)		7 (3.4)		(4.0)	

**Note:**

* *p*-Value from Cochran test.

In the univariate analysis (Tables 4 and 5), we found nine parameters that correlated significantly with the EQ-5D index at 12 months. These parameters included patient age, CCI, Barthel Index, Yesavage Depression Scale, Lawton and Brody Scale, residential status, polymedicated status, previous hip fracture and FAC. The strongest correlations were found with the Barthel Index (Spearman’s Rho coefficient = 0.484), Lawton and Brody (Spearman’s Rho coefficient = 0.502) and Yesavage Depression Scale (Spearman’s Rho coefficient = −0.295).

**Table 4:** Factors associated with EQ-5D Index basal and EQ-5D VAS after 1 year of the surgical intervention.

	EQ-5D index 12 months (*n* = 219)		Difference EQ-5D Index baseline-12 months		EQ-5D VAS 12 months (*n* = 219)		Difference EQ-5D VAS baseline-12 months	
	Rho*	*p*-Value	Rho*	*p*-Value	Rho*	*p*-Value	Rho*	*p*-Value
Age	−0.295	0.000	−0.078	0.269	−0.288	0.000	−0.186	0.008
Time elapsed between hospital admission and intervention	0.020	0.778	−0.035	0.620	0.014	0.841	−0.032	0.654
Time elapsed between hospital admission and discharge	−0.054	0.450	−0.022	0.756	−0.086	0.222	−0.120	0.089
Charlson comorbidity index	−0.198	0.005	−0.016	0.818	−0.160	0.007	−0.069	0.331
Baseline Barthel index	0.484	0.000	−0.278	0.000	0.392	0.000	−0.092	0.192
Baseline Lawton and Brody scale	0.502	0.000	−0.196	0.005	0.451	0.000	0.035	0.618
Baseline Yesavage’s geriatric depression scale	−0.335	0.000	0.067	0.348	−0.45	0.000	0.097	0.171
Baseline Gijon scale (non- institutionalized)	−0.007	0.927	0.050	0.520	0.019	0.808	−0.007	0.924

**Note:**

* Spearman’s Rho correlation.

**Table 5:** Factors associated with baseline EQ-5D index basal and EQ-5D VAS after 1 year of the surgical intervention.

		EQ-5D index 12 months		Difference EQ-5D index 12 months-baseline		EQ-5D VAS 12 months		Difference EQ-5D VAS 12 months-baseline	
		Media	*p*-Valor*	Media	*p*-Valor*	Media	*p*-Valor*	Media	*p*-Valor*
Gender	Male	0.23 (0.35)	0.976	−0.46 (0.36)	0.252	49.8 (19.3)	0.180	−27.0 (16.9)	0.749
	Female	0.22 (0.31)		−0.38 (0.35)		48.8 (16.6)		−28.1 (16.4)	
Living status	Non-institutionalized	0.25 (0.34)	0.003	−0.41 (0.35)	0.435	47.3 (15.9)	0.006	−27.4 (17.2)	0.234
	Institutionalized	−0.19 (0.50)		−0.36 (0.37)		40.3 (8.6)		−29.9 (13.2)	
Polymedicated	No	0.33 (0.35)	0.012	−0.43 (0.37)	0.525	50.6 (17.3)	0.023	−28.6 (16.9)	0.428
	Yes	0.18 (0.30)		−0.39 (0.37)		43.9 (13.6)		−27.5 (16.3)	
Type of surgical intervention	Intramedullary nail	0.19 (0.31)	0.066	−0.43 (0.35)	0.077	45.5 (14.3)	0.541	−27.3 (16.3)	0.477
	Hip replacement (prosthesis)	0.28 (0.34)		−0.33 (0.37)		46.8 (16.4)		−29.0 (17.2)	
Complications	No	−0.05 (0.479)	0.069	−0.40 (0.35)	0.952	46.9 (14.9)	0.058	−27.3 (16.2)	0.224
	Yes	−0.07 (0.44)		−0.40 (0.41)		41.0 (15.7)		−31.4 (18.3)	
Type of fracture	Neck	0.28 (0.34)	0.098	−0.33 (0.36)	0.075	46.9 (16.4)	0.639	−28.5 (16.6)	0.725
	Trochanter	0.19 (0.31)		−0.43 (0.35)		45.7 (14.5)		−27.5 (16.5)	
Previous hip fracture	No	0.24 (0.33)	0.040	−0.39 (0.35)	0.538	46.4 (15.5)	0.512	27.5 (16.7)	0.405
	Yes	0.06 (0.18)		−0.46 (0.41)		43.5 (9.8)		−31.2 (13.5)	
Functional Ambulation Classification (FAC)	Do not walk independently	0.08 (0.24)	0.000	−0.30 (0.35)	0.001	40.9 (11.9)	0.012	−26.4 (14.6)	0.205
	Walk independently	0.32 (0.33)		−0.47 (0.359)		49.4 (15.9)		−29.2 (17.4)	

**Note:**

* U Mann–Whitney.

The prefracture Barthel Index, prefracture Lawton and Brody Scale and prefracture FAC correlated significantly with the difference between the prefracture EQ-5D index and the EQ-5D index at 1 year after surgery, and the correlation coefficients were lower.

The strongest correlation between the EQ-5D VAS score at 1 month was with Lawton and Brody (*r* = 0.451), the prefracture Barthel Index (*r* = 0.392), and the Yesavage Depression Scale (*r* = −0.045) (Table 4).

In the multiple regression analysis (adjusted *R*^2^ = 0.318), the Yesavage Depression Scale baseline (*B* = −0.023 (−0.34 to −0.013), *p* < 0.001) and the patient age (B = −0.011 (−0.18 to −0.04), *p* < 0.002) were significantly associated with a lower EQ-5D index at 1 year after surgery, while the Lawton and Brody baseline (*B* = 0.41 (0.25–0.58), *p* < 0.003) was associated with higher EQ-5D index levels (Table 6). For the difference between the EQ-5D Index just before surgery and 1 year after surgery, the Barthel Index (*B* = −0.006 (−0.009 to −0.003), *p* < 0.001) was identified as an independent variable (adjusted *R*^2^ = 0.077) (Table 7).

**Table 6:** Multiple regression analysis of factors influencing Index EQ-5D at 12 months.

EQ-5D index 12 months	B	β	Confidence interval of 95% to B		*p* Value
(Constant)	0.991		[0.351–1.631]		0.003
Lawton and Brody scale	0.041	0.342	[0.025–0.058]		0.000
Yesavage’s geriatric depression scale	−0.023	−0.266	[−0.34 to 0.013]		0.000
Age	−0.011	−0.209	[−0.018 to 0.04]		0.002

**Notes:**

B, non-standardized regression coefficients.

β, standardized regression coefficients.

R2 Adjusted, 0.318.

**Table 7:** Multiple regression analysis of factors influencing difference between index prior to fracture and at 12 months.

EQ-5D index difference baseline-12 months	B	β	Confidence interval of 95% to B		*p* Value
(Constant)	0.156		[−0.112 to 0.423]		0.252
Barthel index	−0.006	−0.286	[−0.009 to −0.003]		<0.001

**Notes:**

B, non-standardized regression coefficients.

β, standardized regression coefficients.

R2 adjusted, 0.077.

In the multiple regression analysis of factors influencing the EQ-5D VAS score at 1 year (adjusted *R*^2^ = 0.257), a higher presurgery Lawton and Brody score was associated with a higher EQ-5D VAS (*B* = 1.683 (0.914–2.451), *p* < 0.001), while the Yesavage Depression Scale (*B* = −1.184 (−1.688 to −0.680), *p* = 0.001) and the patient age (*B* = −0.340 (−0.670 to −0.10), *p* = 0.002) were associated with lower EQ-5D VAS levels (Table 8). Regarding the difference between the EQ-5D Index just before surgery and at 1 year after surgery, the patient age (*B* = −0.518 (−0.902 to −0.134), *p* < 0.008) was identified as an independent variable (adjusted *R*^2^ = 0.029) (Table 9).

**Table 8:** Multiple regression analysis of factors influencing EQ 5D VAS at 12 months.

EQ-5D VAS 12 months	B	β	Confidence interval of 95% to B		*p* Value
(Constant)	67.880		[37.342–98.418]		<0.001
Lawton and Brody scale	1.683	0.300	[0.914–2.451]		<0.001
Baseline Yesavage’s geriatric depression scale	−1.184	−0.297	[−1.688 to −0.680]		0.001
Age	−0.340	−0.138	[−0.670 to −0.10]		0.002

**Notes:**

B, non-standardized regression coefficients.

β, standardized regression coefficients.

R2 adjusted, 0.257.

**Table 9:** Multiple regression analysis of factors influencing difference between EQ-5D VAS prior to fracture and at 12 months.

EQ-5D VAS Difference 12 Months-baseline	B	β	Confidence interval of 95% to B		*p* Value
(Constant)	15.880		[−16.612 to 48.373]		0.336
Age	−0.518	−0.185	[−0.902 to −0.134]		0.008

**Notes:**

B, non-standardized regression coefficients.

β, standardized regression coefficients.

R2 adjusted, 0.029.

## Discussion

Hip fractures are the most frequent cause of admission to trauma units for older people, which often results in reduced mobility and a loss of independence. One of the goals of our study was to employ several methods to statistically demonstrate the impairment in the HRQOL: the EQ-5D questionnaire (VAS and index score) and the SF-12 health survey (PCS-12 and MCS-12). Most studies designed to evaluate the HRQOL have employed the EQ-5D index score (social tariff). However, this score has limitations, such as a bimodal or trimodal distribution or a ceiling effect, and it does not provide information on the change in HRQOL (Ostendorf et al., 2004). Another goal of our study was to separately evaluate the distribution of the EQ-5D dimensions.

The use of the HRQOL, as a tool for understanding the specific effect of a condition (in our case, hip fracture) on the life of the patient, has increased significantly in recent decades. However, to our knowledge, there is still limited information on the quality of life after hip fracture in the Spanish population in the medium and long term. An international study (Borgström et al., 2013) with data from 11 countries evaluated the quality of life at 4 months after hip fracture using the EQ-5D questionnaire, and the Spanish section consisted of 46 patients. The multicenter study on (Caeiro et al., 2017) the quality of life 12 months post hip fracture (Martínez et al., 2014), evaluated the quality of life related to health in patients with subcapital fracture of femur subjected to different hemostatic treatments, where the EQ-5D scale was used with five levels of severity, while the Ubeda study (Úbeda et al., 2015) evaluated the quality of life in patients with hip arthroplasty secondary to osteoarthritis, where the fracture was an exclusion criterion.

The population of our study mainly consists of women older than 80 years who have a low index of institutionalization and that are consistent with the other studies. (Martínez et al., 2014; Úbeda et al., 2015; Ramírez-Pérez et al., 2014; Milte et al., 2018; Parsons et al., 2018; Moerman et al., 2016; Tidermark et al., 2003; Sugeno et al., 2008). The length of hospital stay was 5.3 days (±1.2), which is considerably less than that shown in other studies (Úbeda et al., 2015) and (Caeiro et al., 2017) at 11.8 days (±7.9). Notably, there was a 21% increase in the rate of institutionalization of elderly individuals who suffered a hip fracture at 12 months.

Mortality from all causes a year after the fracture is lower than that evidenced in the study by (Caeiro et al., 2017) (8.5 vs 15.8), although the comorbidity index was much higher in our study (5.3 ± 1.2 vs 1.9 ± 1.3). In the study of (Ruths et al., 2017), using the ASA risk as an indirect indicator of comorbidity, the results were very similar to those of our study, (ASA III—60.7% vs. 53%) (ASA IV 7.6% vs 6.7%), although mortality was much higher (25.3%).

It is necessary to indicate that at the time of hospital admission, our patients had an adequate level of functional independence (Barthel index = 87.6), an adequate social-familiar situation (Gijon scale = 8.3), and only 16.5% were institutionalized. The indices that assess the motor abilities and functional performance experienced a decline in the first month, but in successive evaluations at 6 and 12 months, these rates experienced a slight increase or stabilization. This trend was not fulfilled in the Yesavage index, where the index at 12 months exceeds the results found 1 month after the intervention by one point. The patients in our study had a capacity to develop the BADL, in comparison with the study of (Caeiro et al., 2017), Barthel 87.6 (±16.9) vs 77.5 (±26.9), although after 12 months, there was no improvement at levels similar to those before the fracture; however, this improvement occurred in several studies (Caeiro et al., 2017; Sugeno et al., 2008).

The baseline values of the EQ-5D index show an acceptable self-assessment of quality of life, similar to that provided by other studies conducted in the Spanish population (Caeiro et al., 2017; Borgström et al., 2013; Ferrer et al., 2015), and studies conducted in different countries, such as the United Kingdom (Parsons et al., 2018), Estonia (Jürisson et al., 2016), Thailand (Amphansap & Sujarekul, 2018) and Mexico (Guirant et al., 2018), although somewhat lower than that reported by other studies in Japan (Sugeno et al., 2008) and Australia (Abimanyi-Ochom et al., 2015) and in the systematic review by Peasgood et al. (2009).

Although when evaluating each of the domains independently, the high affectation of the domains of mobility, pain or discomfort and daily activities is noteworthy, where more than 50% of the patients reported having problems in development, contrary to the results of (Gjertsen et al., 2016), although the mean age of the population in their study was considerably lower (84.6 ± 6.1 vs 77.3 ± 11.7). Our data regarding mobility and self-care activities before the fracture are in line with those obtained by (Guirant et al., 2018), where these authors evaluated the changes in HRQOL (by using EQ-5D) during 12 months in 193 patients with hip fracture, and the population showed higher basal levels of anxiety and depression. When comparing the data after 12 months, we observed that the population of our study was most greatly affected in all domains, with the domain showing greater difference with respect to the basal levels of self-care (Milte et al., 2018). A comparison of two tools to measure HRQOL (EQ-5D and ICECAP-O) in patients with hip fracture showed a significant deterioration in HRQOL at 4 months after fracture. The majority of the patients indicated problems with mobility, self-care, usual activities and pain/discomfort (information the EQ-5D dimensions). In addition, approximately one in two individuals experienced moderate or severe anxiety or depression. Moreover, most of our patients reported experiencing some problem with each of the dimensions of the EQ-5D at 12 months after hip fracture, and 32.7% and 65.8% of patients reported severe problems in self-care and usual activities, respectively. These findings agree with those described in another study (Kondo et al., 2014)

Notably, in the studies of (Parsons et al., 2018) and (Sugeno et al., 2008), the evaluation of the EQ-5D VAS showed baseline values lower than those provided in our study (67.6 and 62.6), but much higher values than those of our studies in the follow-ups ((Parsons = 67.6 basal, 33.2 at 6 months and 66.9 at 12 months) (Sugeno = 62.6 basal; one a month and 79.6 at 12 months). On the other hand, the study of Amphansap T (2018) showed higher initial values (85) but a marked decrease at 3 months (43), although these values increased significantly in months 6 to 12 (68 and 79).

Regarding the evolution of the EQ-5D index, most of the studies showed a rapid decline in short-term scores (hospital discharge or 1 month after fracture) and an improvement in the values at 12 months with respect to midterm evaluations (4 months) (Parsons et al., 2018; Sugeno et al., 2008; Amphansap & Sujarekul, 2018; Guirant et al., 2018; Abimanyi-Ochom et al., 2015; Jürisson et al., 2016; Campenfeldt et al., 2017; Støen et al., 2014; Frihagen, Nordsletten & Madsen, 2007; Keating et al., 2006), except for our study, which showed a stabilization of the values at 6–12 months of 0.3 (SD ± 0.2). This finding could be explained by the differences in the cut-off midterm follow-up, which was carried out in our study at 6 months postfracture, while in the majority of the studies this evaluation was performed at 4 months. It is possible that even the recovery margin was wider than the 6-month record.

In the study developed by (Kelly-Pettersson et al., 2019) aimed at examining the influence of depression on quality of life at 1 year after a hip fracture, with follow-up evaluations at 3 and 12 months, the scores at both 3 and 12 months in the control group and the group of patients with depression were somewhat lower than the baseline scores, similar to the findings in the study of (Tidermark et al., 2002), where the HRQOL of patients over 65 years of age deteriorated significantly at 4 and 17 months after a femoral neck fracture. When using the EQ-5D questionnaire, the authors revealed a significant decrease of 0.78 in the baseline to 0.59 and 0.51 in postfracture follow-ups.

Moerman et al. (2016) reported a significant decrease in HRQOL using the SF-12 health survey in their work between the beginning and after 3 months of hip fracture. The values of MCS recovered to baseline levels after 12 months, whereas the PCS did not. In our study, both components were far from their initial values at both 6 and 12 months, and even the MSC component reduced its value with respect to 6 months. (Tseng et al., 2016) evaluated the quality of life at month, 3, 6 and 12 months after hip fracture using the abbreviated questionnaire SF36. The average PCS score was 45.53 (±5.92) at 1 month after discharge and improved to 63.67 (±10.88) at 12 months after discharge. The MCS score was 55.31 (±9.72) at 1 month after discharge and remained relatively stable during the first 3 months after discharge but decreased slightly to 51.97 (±9.53) at 12 months after discharge. All values were superior to those reported in our study.

The univariate analysis showed a correlation of the HRQOL with age, ICC, BADL, IADL, depression, previous hip fracture, baseline living status, polymedicated status and walk independently. The multivariate analysis confirmed the correlation between HRQOL and BADL, IADL, age and depression. For the EQ-5D VAS, we found similar results to the univariate analysis but did show a correlation with previous hip fracture. The multivariate analysis found correlations with BADL, depression and age.

Moerman et al. (2016) also identified factors potentially associated with the decline in HRQOL (in PCS, not in MCS): age over 80, treatment with osteosynthesis, higher prefracture level of mobility, intracapsular fracture, and ASA classification I and II. (Suwanpasu & Pongpaew, 2016) indicated that HRQOL could be improved in cases of identifying (and treating) limitations in daily living activity, frailty, and depression. In our study, a better HRQOL was associated with not having depression after 12 months, walking independently after 12 months, a higher HRQOL (EQ-5D VAS) at baseline, and younger ages.

The HRQOL at baseline has been associated with the first incident of hip fracture in postmenopausal women (Chen et al., 2018). The authors demonstrated that at baseline, the scores for the SF-36 questionnaire in women who suffered a hip fracture were significantly lower than those for the controls (same age but no hip fracture). Similarly, the HRQOL at baseline was the main factor contributing to the loss in HRQOL after 12 months, as demonstrated by Guirant et al. (2018).

The result of a recent systematic review (Peeters et al., 2016) showed that HRQOL and health status are negatively associated with female gender, comorbidity, inadequate nutritional status, low physical or psychosocial functioning prior to hip fracture, longer hospital stays, postoperative complications and pain.

### Limitations

One limitation of this study is the impossibility of obtaining information prospectively on the prefracture situation and therefore assuming the possibility of memory bias and underestimation of the results (Scholten et al., 2017).

Another limitation of the present study is the use of a non-probabilistic sampling technique, although the study has been carried out through consecutive sampling, including all those patients who during the study period met the requirements for their participation, this type of Sampling, while not ensuring the representativeness of the entire population, is the most approximate method.

The use of the EQ-5D tool with only three levels of severity may have a low discriminatory power compared to tools that use higher levels of severity.

The HRQOL of patients after hip fracture may be influenced by other unrelated factors, such as pre-existing comorbidities (Polinder et al., 2010). Although we are aware of this limitation, it is intrinsically linked to studies aimed at evaluating the impact of certain pathologies on HRQOL. As with most studies, our point of reference was the baseline situation 2 weeks before the fracture, on the understanding that the memory bias, if any, would be minimal since the survey is conducted at the time of admission to the hospital. In addition, our multivariate analysis ruled out comorbidities (Charlson Index) at the time of admission as an independent factor associated with HRQOL. Another limitation of the study was the impossibility of determining whether the changes identified were a direct cause of the hip fracture or were influenced by other vital situations that occurred during the study period.

## Conclusions

Patients experience a significant impairment in all dimensions of the HRQOL after a hip fracture in all domains, especially self-care, usual activities and mobility. The decline in the HRQOL was more marked in the first month and increased slightly as the recovery progressed; however, these values was very low from the initial values at 12 months after the surgical intervention. Further studies involving a larger cohort of patients, control groups and longer prospective follow-up periods are required to corroborate the present results.

## Supplemental Information

10.7717/peerj.9215/supp-1 Supplemental Information 1 Raw data.

10.7717/peerj.9215/supp-2 Supplemental Information 2 Codebook- raw data.

## List of Abbreviations

ASA: American Society of Anesthesiologists
BI: Barthel Index
EQ-5D: EuroQol-5 Dimensions
EQ-5D 3L: EuroQol-5 Dimensions 3 Levels
HRQOL: Health-related quality of life
IQR: The Interquartile Range
SF 12: Health Survey
SD: Standard Deviation
VAS: Visual Analog Scale

## Additional Information and Declarations

### Data Availability

The following information was supplied regarding data availability: The raw measurements are available in a Supplemental File.
