# Supplementary material for: Quality of life after hip fracture: a 12-month prospective study
Source: PeerJ. 2020 Jun 16;8:e9215. doi: 10.7717/peerj.9215 (PMC7304420; doi:10.7717/peerj.9215)
Supplement: Supplemental Information 2 [file peerj-08-9215-s002.docx]

| HOSPITAL | |  | |  | | Coexistence | |  | |
| --- | --- | --- | --- | --- | --- | --- | --- | --- | --- |
| HSPA | | 0 | |  | | live alone | | 0 | |
| HCA | | 1 | |  | | live as a couple | | 1 | |
| Destination_hospital_discharge | |  | |  | | live with family /friends | | 2 | |
| Own address | | 0 | |  | | supervised flat | | 3 | |
| INSTITUTION | | 1 | |  | | nursing home | | 4 | |
| Other (family) | | 2 | |  | | Monthly income (euros) | |  | |
| Exitus | | 3 | |  | | less500 € | | 0 | |
| AGE | |  | |  | | 500€-1000€ | | 1 | |
| AGE GROUP | |  | |  | | 1000 €-1500€ | | 2 | |
| 65-70 | | 0 | |  | | > 1500 € | | 3 | |
| 70-75 | | 1 | |  | | Profession | |  | |
| 75-80 | | 2 | |  | | housewife | | 0 | |
| 80-85 | | 3 | |  | | farming | | 1 | |
| 85-90 | | 4 | |  | | operator | | 2 | |
| > 90 | | 5 | |  | | official | | 3 | |
| SEX | |  | |  | | housewife and other activity | | 4 | |
| MALE | | 0 | |  | | SEASON OF THE FALL | |  | |
| FEMALE | | 1 | |  | | summer | | 0 | |
| EDUCATION LEVEL | |  | |  | | autumn | | 1 | |
| No studies | | 0 | |  | | winter | | 2 | |
| Primary studies | | 1 | |  | | spring | | 3 | |
| Bachelor | | 2 | |  | | PLACE OF THE FALL | |  | |
| University studies | | 3 | |  | | street /public via | | 0 | |
| Civil status | | | |  | | own address | | 1 | |
| single | | 0 | |  | | home | | 2 | |
| married | | 1 | |  | | others (other home, shops) | | 3 | |
| separated | | 2 | |  | |  | |  | |
| widow | | 3 | |  | |  | |  | |
|  | |  | |  | |  | |  | |
| fracture type | | |  | | treatment (0=no 1=yes) | | | |  |
| Neck | 0 | |  | | psychotropic | |  | |  |
| Troncanterics | 1 | |  | | bisphophonates | |  | |  |
| surgical treatment | | |  | | calcium and vitamin d | |  | |  |
| No | 0 | |  | | antiplatelets | |  | |  |
| Yes | 1 | |  | | anticoagulants | |  | |  |
| Type intervention | | |  | | heparin | |  | |  |
| Intramedullary nail | 0 | |  | | number of medications | | | |  |
| hip prothesis (unipolar, bipolar y total) | 1 | |  | | 0 | | 0 | |  |
| cannulated screws | 2 | |  | | 0-5 | | 1 | |  |
| Type of anesthesia |  | |  | | 5 A 10 | | 2 | |  |
| GENERAL | 0 | |  | | > 10 | | 3 | |  |
| Spinal | 1 | |  | | Polymedicated (>=5) | | (0=no 1=yes) | |  |
| Spinal with sedation | 2 | |  | | alterations of the sensory system (vision / hearing) | | | |  |
| Nervous lock | 3 | |  | | yes | | 1 | |  |
| complications after surgical intervention |  | |  | | no | | 0 | |  |
| No | 0 | |  | | surgical risk ASA | |  | |  |
| yes | 1 | |  | | no reflected | | 0 | |  |
| what complications? |  | |  | | ASA I | | 1 | |  |
| None | 0 | |  | | ASA II | | 2 | |  |
| periclave fracture, Nail loosening, ,prosthesis luxation | 1 | |  | | ASA III | | 3 | |  |
| surgical wound infection | 2 | |  | | ASA IV | | 4 | |  |
| pulmonary embolism | 3 | |  | | ASA V | | 5 | |  |
| deep venous thombosis | 4 | |  | | depressive mood | |  | |  |
| confusional syndrome | 5 | |  | | yes | | 1 | |  |
| will not be able to support | 6 | |  | | no | | 0 | |  |
| exitus | 7 | |  | | body mass index | |  | |  |
| personal history (0=no 1=yes) | | |  | | normopeso | | 0 | |  |
| arterial hypertension |  | |  | | overweight | | 1 | |  |
| Diabetes Mellitus |  | |  | | obesity | | 2 | |  |
| dyslipidemia |  | |  | |  | |  | |  |
| Osteoporosis |  | |  | |  | |  | |  |
| previous hip fracture |  | |  | |  | |  | |  |

SCALES

| **barthel index** | **dependence** | code |
| --- | --- | --- |
| <20 | total | 0 |
| 20-35 | serius | 1 |
| 40-55 | moderate | 2 |
| 60-95 | mild | 3 |
| 100 | independent | 4 |

| **Lawton & Brody index** | male | female | code |
| --- | --- | --- | --- |
| total dependece | 0 | 0 - 1 | 0 |
| serius dependence | 1 | 2 y 3 | 1 |
| moderate dependece | 2 Y 3 | 4 y 5 | 2 |
| mild dependence | 4 | 6 y 7 | 3 |
| independent | 5 | 8 | 4 |

| **Social Scale Gijon index** |  |  | code |
| --- | --- | --- | --- |
| social risk | ≥16 | YES | 1 |
| no social risk | ≤15 | NO | 0 |
| residence/institution |  |  | 20 |

| **CHARLSON Index** |  | code |
| --- | --- | --- |
| comorbility absence | 0-1 | 0 |
| low comorbility | 2 | 1 |
| high comorbility | ≥ 3 | 2 |
